# Low levels of ATM in breast cancer patients with clinical radiosensitivity

**DOI:** 10.1186/2041-9414-1-9

**Published:** 2010-06-24

**Authors:** Zhiming Fang, Sergei Kozlov, Michael J McKay, Rick Woods, Geoff Birrell, Carl N Sprung, Dédée F Murrell, Kiran Wangoo, Linda Teng, John H Kearsley, Martin F Lavin, Peter H Graham, Raymond A Clarke

**Affiliations:** 1 Department of Radiation Oncology, St George Clinical School of Medicine University of NSW, St George Hospital, Kogarah, NSW 2217, Australia; 2 Queensland Institute of Medical Research Herston, Queensland 4006, Australia; 3 Australian National University and The Canberra Hospital Department of Radiation Oncology, Building 3, Level 1, Garran, A.C.T. Australia; 4 Centre for Women's Health Research, Monash Institute of Medical Research, Monash University, Clayton, Victoria, Australia; 5 Department of Dermatology, St George Clinical School of Medicine University of NSW, St George Hospital, Kogarah, NSW 2217, Australia

## Abstract

**Background and Purpose:**

Adjuvant radiotherapy for cancer can result in severe adverse side effects for normal tissues. In this respect, individuals with anomalies of the ATM (ataxia telangiectasia) protein/gene are of particular interest as they may be at risk of both breast cancer and clinical radiosensitivity. The association of specific ATM gene mutations with these pathologies has been well documented, however, there is uncertainty regarding pathological thresholds for the ATM protein.

**Results:**

Semi-quantitative immuno-blotting provided a reliable and reproducible method to compare levels of the ATM protein for a rare cohort of 20 cancer patients selected on the basis of their severe adverse normal tissue reactions to radiotherapy. We found that 4/12 (33%) of the breast cancer patients with severe adverse normal tissue reactions following radiotherapy had ATM protein levels < 55% compared to the mean for non-reactor controls.

**Conclusions:**

ATM mutations are generally considered low risk alleles for breast cancer and clinical radiosensitivity. From results reported here we propose a tentative ATM protein threshold of ~55% for high-risk of clinical radiosensitivity for breast cancer patients.

## Background

Primary treatment for organ-confined breast cancer usually involves a combination of limited breast-conservation surgery and radiotherapy yielding control rates equivalent to mastectomy. Such radiotherapy is usually tolerated well except for the very small percentage of patients who experience severe adverse normal tissue reactions (RTOG3-4 reactions) [1]. There is little empirical evidence to support anecdotal reports of clinical radiosensitivity in connective tissue disorders including systemic lupus erythematosus (SLE) [2-5]. However, other genetic predispositions, including autosomal recessive ataxia telangiectasia (A-T), have a clear association with clinical radiosensitivity [6,7]. A-T is an autosomal recessive disorder characterised by early-onset neurodegeneration, oculocutaneous telangiectasia, immunodeficiency, hypogonadism, cancer susceptibility and acute hypersensitivity to radiotherapy [6]. ATM, the susceptibility gene for A-T, encodes a protein kinase activated in response to ionising radiation (IR) induced DNA double-strand breaks that facilitates the phosphorylation of numerous molecular intermediates involved in cell-cycle regulation and DNA repair. A-T patients usually harbour compound ATM mutations comprising either two non-allelic null mutations or one null and one pathogenic missense mutation [7,8].

The approximately 1% of individuals in the general population which are A-T heterozygotes [9,10] do not present with any of the more severe early-onset A-T pathologies, however, they are considered at risk of breast cancer [9,11,12] and/or clinical radiosensitivity [9]. Albeit, the level of risk associated with ATM mutations is uncertain. In 2002 Inannuzzi et al [9] found that a small cohort of clinically radiosensitive breast cancer patients had a higher incidence of ATM missense variants; unfortunately, no functional deficit was established for any of these variants in the form of reduced levels of either ATM protein or ATM kinase activity. In the same year, we described the first heterozygous ATM missense mutation causing dominant negative effects in breast cancer including cellular radiosensitivity and reduced ATM kinase activity but no obvious reduction in the level of the ATM protein [10]. However, not all missesnse variants are associated with dominant negative effects. By comparison, individuals heterozygous for one of the many reported ATM null mutations have reduced levels of the ATM protein, albeit, these reduced levels do not always appear to fall below the threshold for risk of breast cancer and/or clinical radiosensitivity [8,10,12-18]. For want of improving risk assessments for breast cancer and radiosensitivity it would therefore be advantageous to establish pathological threshold levels for the ATM protein.

In the present study we used comparative immuno-blotting of the ATM protein to investigate a cohort of cancer patients selected on the basis of their severe adverse reactions to adjuvant radiotherapy and compared these with control patients with no adverse reactions. We also compared ATM levels for a group of women with SLE [19]. The radiosensitive breast cancer patients in our cohort expressed a much wider range of ATM protein levels compared with the non-reactor control group. Patients with low ATM levels were evaluated further for ATM kinase activity, IR-induced chromosomal aberrations (ICA) and ATM mutation status using RT-PCR and genomic sequencing. For those individuals with multiple ATM mutations we established allelic status. Results suggest that ATM protein levels are useful for identifying breast cancer patients at high-risk of clinical radiosensitivity.

## Methods

We obtained human ethics approval from the Institutional Review Boards of the three institutions involved in this study. We obtained informed consent from paticipants in accordance with the guidelines set down by the National Health and Medical Research Council of Australia. In should be appreciated here that very severe reactions to radiotherapy are particularly rare entities and that it was only through close personal communication between 3 of Australia's major cancer centres that we were able to assemble a cohort of 20 cancer patients that had had very severe adverse normal tissue reactions: The Cancer Care Centre, St George Hospital, Sydney; Queensland Radium Institute, Brisbane and Peter MacCallum Cancer Institute, Melbourne, Australia. Courses of radiotherapy were uniformly delivered with 3-dimensional (3D) conformal techniques using a linear accelerator (4 MV/6 MV) at scheduled doses of 46 Gy in 20 fractions to the breast with a boost of 10 Gy in five fractions to the primary tumour excision site or 50 Gy in 25 fractions to the chest wall post mastectomy. These patients were not otherwise part of a clinical trial or any other organised protocol. Acute toxicity was defined as toxicity from the time of commencement of radiotherapy through to the 90^th ^day after treatment and late toxicity between 90 days and 5 years and assessment was based on the RTOG grading system [20]. Severe acute or late toxicity was defined as ≥ grade 3.

### 2.1 Participants

Peripheral venous blood samples were collected from 52 consenting volunteers including 20 individuals with severe adverse normal tissue reactions to radiotherapy, for cancer of the cervix (2), prostate (6) and breast (12). We also included breast cancer patients with no adverse reactions (14); 11 women with SLE one of whom had had previous treatment for breast cancer with no adverse reaction to radiotherapy; 2 individuals with A-T and 5 parents of A-T individuals (A-T obligate heterozygotes), 4 of whom had ATM null-type mutations (see results). We generated lymphoblastoid cell lines (LCLs) from each paticipant. LCLs were maintained in RPMI 1640 medium supplemented with 10% foetal bovine serum and 1% l-glutamine in a 5% CO_2 _atmosphere at 37°C.

Follow-up examinations were performed on a 3-4-month basis for the 1^st ^2 years and 6 monthly for the 3^rd ^and 4^th ^years followed by a final appointment in the 5^th ^year. A retrospective chart reviewing all radio-hypersensitive patients was prepared, and morbidity data was collected from the follow-up notes by a radiation oncologist who was unaware of the research results.

### 2.2 Comparative Western analysis of ATM protein levels

#### Antibodies

We used the ATM 2C1 monoclonal antibody (Abcam/GeneTex) for ATM Western analysis and Kinase assays. The constitutive level of the ATM protein for LCLs was determined by western analysis using 4% polyacrylamide gel electrophoresis as described previously [15]. Cell extracts were initially denatured in SDS buffer (58 mM TrisHCl, pH 6.8, 1.71% SDS (w/v), 0.83% (w/v) β-mercaptoethanol, glycerol 6% (v/v), 0.002% (w/v) bromophenol blue) at 100°C for 5 min and loaded onto a 4% SDS PAGE gel. After electrophoresis proteins were transferred to nylon membranes (1 hour) using a semi-dry blotting apparatus (BioRad) and then stained with Ponseau-S to confirm comparative protein loading and transfer efficiency. Blots were washed in TBS-tween20 (TBS-T) and then blocked overnight at 4°C with 5% skim milk in TBS-T.

Membranes were incubated with appropriate primary antibodies diluted in 5% skim milk (ATM-2C1 primary was used at 1:2,000 and the GAPDH primary antibody was used at 1:15,000) in TBS for 1 hour, followed by 5 × washes with TBS-T at room temperature and then incubated with the appropriate secondary antibody conjugates (HRP conjugated (Sigma) diluted 1:20,000) in 10% skim milk in TBS for 30 min at room temperature, followed by five washes with TBS-T at room temperature. Equivalent protein loading (50 μg) was confirmed for each sample by comparing Ponseau S staining patterns and by comparing immunoblot signals for a second protein ~ glyceraldehyde phosphate dehydrogenase (GAPDH) prior to probing with the ATM 2C1 antibody. Western blots were imaged and analysed using a BioRad imaging system. The individual with the highest level of ATM protein was selected as the reference sample termed the 'highest expresser'. Band densities from repeat experiments were averaged for each individual and expressed as a percentage of the highest ATM expresser similar to an earlier study by Richard Gatti's group [16]. The effect of IR on ATM protein levels was similarly determined before and 1 hour after exposure to IR (2 Gy) delivered by a linear accelerator [15,17]. We used the Student's two-tailed t-test to determine the significance of the findings.

### 2.3 In vitro ATM kinase assays

ATM protein kinase assays using GST-p53 as substrate and ATM autophosphorylation assays were performed essentially as described previously [18].

### 2.4 IR-induced chromosome aberrations

IR-induced chromosomal aberration (ICA) counts were performed using an established technique described previously [21]. Cells were irradiated with 1 Gy of IR. For G_2_-phase cells, colcemid at a final concentration of 100 ng/ml was added immediately after irradiation, 1 hour before harvesting. The cells were treated for 15 min with 0.075 M KCl, fixed in methanol-glacial acetic acid (3:1) and spread on glass slides. The cells were then stained with Giemsa and 50 metaphases were analysed for each sample. The number of ICA that persisted 1 hour after exposure to IR was reported as average aberrations per metaphase.

### 2.5 Cell-growth delay assays after in vitro exposure to IR

Cell-doubling times were calculated prior to radiation experiments. Using pre-conditioned medium, aliquots of each LCL in log-phase were plated in preconditioned media in 7 cm flasks at a density of approximately 5 × 10^4 ^cells/mL. Cells were incubated for 24 hours prior to irradiation with 2 Gy. Radiation was delivered by a 6-MV linear accelerator with beam quality TPR2010 = 0.68 at a high dose rate of approximately 2.5 Gy/min. Cell-growth delay (SF_2_) values were estimated relative to non-irradiated control cell lines after four doubling times as the average of three repeat experiments. Mean values for cohorts were expressed as the cell-growth delay value ± the standard error (SD/vn).

### 2.6 Nucleotide sequence analyses

Total RNA and genomic DNA were isolated from LCLs using TRI-Reagent (Sigma Aldrich T9424). cDNA was reverse transcribed using SuperScript III (Invitrogen 18080-044) and 500 ng of total RNA as template. The full ATM transcript was PCR amplified as a series of 8 overlapping fragments as described previously [22-24]. PCR amplicons were electrophoresed on a 1% agarose gel then excised and purified using the Perfectprep Gel Cleanup Kit (Eppendorff) and subjected to forward and reverse sequencing using the original PCR primer sets. Sequence analysis was preformed using BioEdit Software http://www.mbio.ncsu.edu/BioEdit/bioedit.html.

Intron/exon boundaries and variant nomenclature were determined relative to RefSeq NM_000051 on the LOVD ATM variant listings on web site http://chromium.liacs.nl/lovd/refseq/ATM_codingDNA.html. DNA mutation numbering was based on the cDNA sequence, where +1 corresponds to A of the ATG translation initiation codon. The initiation codon is counted as codon 1.

## Results

### 3.1 Patients and Radiotherapy

Patients with cancer of the breast, prostate and cervix (reviewed in Table 1) were selected solely on the basis of their severe adverse normal tissue reactions to radiotherapy (RTOG3-4). Breast cancer patients 10, 12 and 13 had relatives also diagnosed with breast cancer (Table 1). Severe late normal tissue reactions were observed in 18/20 cancer patients and for 2 of these (patients 10 & 11) the late reaction was preceded by a severe acute reaction. Another 2 patients, patients 1 and 9, exhibited severe acute reactions only.

**Table 1:** Clinical review for patients with adverse normal tissue reactions to radiotherapy

Patient	Cancer	Sex	ATM%	Age	Reaction	Clinical features
1	Prostate	M	69	64	A	RTOG4 - treatment terminated at 28 Gy
2	Prostate	M	80	72	L	RTOG3 - Proctitis
3	Prostate	M	90	69	L	RTOG3 - Proctitis
4	Prostate	M	82	71	L	RTOG3 - Proctitis
5	Prostate	M	79	60	L	RTOG3 - Cystitis
6	Prostate	M	80	65	L	RTOG3 - Proctitis
7	Cervix	F	71	36	L	RTOG3 - Urethral stricture
8	Cervix	F	81	47	L	RTOG3
9	Breast	F	80	51	A	RTOG3 - Moist desquamation
10*¥∞	Breast	F	35	64	A&L	RTOG4 - Familial Treatment terminated at 20 Gy
11*£¥	Breast	F	45	54	A&L	RTOG3 - Moist desquamation; skin atrophy/pigment
12	Breast	F	82	57	L	RTOG3 - Familial
13*∞£¥	Breast	F	8	50	L	RTOG4 - Familial, skin atrophy & fibrosis, necrosis
14	Breast	F	77	41	L	RTOG3 - Fibrosis (skin, subcutaneous tissue & breast)
15	Breast	F	66	71	L	RTOG4 - Familial
16*£	Breast	F	30	77	L	RTOG4
17	Breast	F	64	47	L	RTOG3
18	Breast	F	88	58	L	RTOG3
19	Breast	F	69	62	L	RTOG3
20	Breast	F	66	70	L	RTOG3

ATM% ~ percentage of ATM protein compared with the highest ATM expresser; * ~ low level of ATM protein < 50% cut-off;

A ~ severe acute reaction; L ~ severe late reaction; ¥ ~ Intermediate ICA count; ∞ ~ ATM mutation(s); £ ~ reduced ATM kinase activity.

### 3.2 ATM Protein Levels

**Figure 1:**
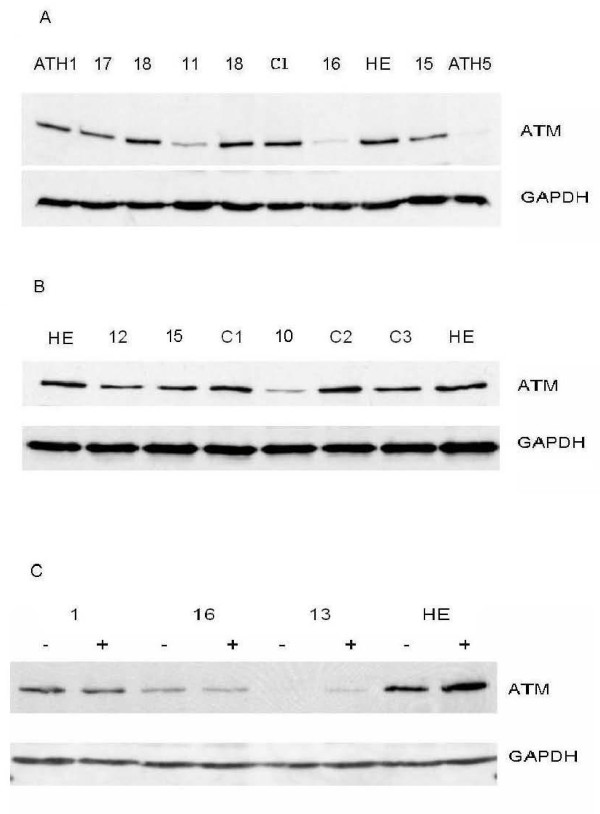
**Western analysis of protein extracts from LCLs established from patients listed in Table**. 1. **A & B**. Basal levels of the ATM protein. **C**. ATM protein levels before and 1 hour after an IR exposure of 5 Gy. Western blots were probed with the ATM-2C1 antibody (Abcam). Obligate A-T heterozygote (ATH); Highest ATM expresser (HE); Non-reactor controls (C1-3).

**Figure 2:**
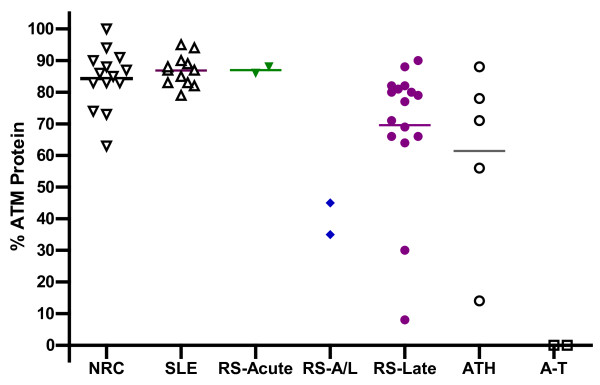
**Scatterplot showing the level of the ATM protein for LCLs established for the following population groups**. Breast cancer patients with no adverse reaction to radiotherapy referred to as non-reactor controls (NRC); Women with SLE; Cancer patients with severe acute normal tissue reactions to radiotherapy (RS-acute); Patients with severe acute and late reactions (RS-A/L); Patients with only severe late reactions (RS-Late); Obligate A-T heterozygotes (ATH - Note: the 4 highest expressing ATH carry null mutations); A-T patients (A-T). ATM protein densities were plotted as a percentage of the level of the ‘highest ATM expresser’.

We determined ATM protein levels from LCLs established from patients: 52 individuals in total. We referred to the individual with the highest level of ATM protein in cell extracts as the 'highest expresser'. The highest expresser was subsequently included as a reference lane in repeat immunoblots (Fig. 1A lane 8, Fig. 1B lanes 1 & 8, Fig. 1C lanes 7 & 8). At least two different cell extracts were analysed and averaged as a percentage of the highest expresser using scatter plots (Fig. 2). The 14 breast cancer patients which had no adverse reaction to radiotherapy, referred to here as 'non-reactor controls' (NRC see Fig. 2), had a mean level of the ATM protein of 84% compared with the highest expresser (range 63% - 100%). For the 2 A-T null cell lines we observed no ATM expression [25]. For the 4 obligate A-T heterozygotes (ATH) with null mutations the ATM protein levels were > 55% (range 56% - 84%) compared to the mean for non-reactor controls (Fig. 2). Unexpectedly, ATH5ABR, the A-T heterozygote control with a missense mutation (see below) was the lowest expresser of all obligate heterozygotes with an ATM protein level ~14% compared to the highest expresser and ~17% compared with the mean for non-reactor controls (Fig. 2 and see also Fig. 1A lane 10). Women with SLE (Fig. 2) had ATM levels > 70% compared with the highest expresser and a mean ATM protein level (87%) comparable to the mean for non-reactor controls (84%) (Fig. 2).

Four of the twelve breast cancer patients with severe late adverse normal tissue reactions to radiotherapy, patients 10, 11, 13 and 16, had levels of ATM protein ~35%, ~45%, ~8% and ~30%, respectively, compared to the highest expresser. These levels of the ATM protein were all < 55% compared with the mean for non-reactor controls (p = 0.0048) (Fig. 2 and see also Fig. 1A lanes 4 & 7, Fig. 1B lane 5 and Fig. 1C lanes 5 & 6). The adverse late reactions to radiotherapy manifest by two of these 'low expressers', patients 10 and 11, were preceded by severe acute affects (Table 1). We over-exposed western blots and found no noticeable expression of any novel ATM truncation products that might otherwise ameliorate or compound the detrimental effects of the low levels of ATM expression [26]. This result was consistent with other studies reporting instability of truncated proteins [27].

**Figure 3:**
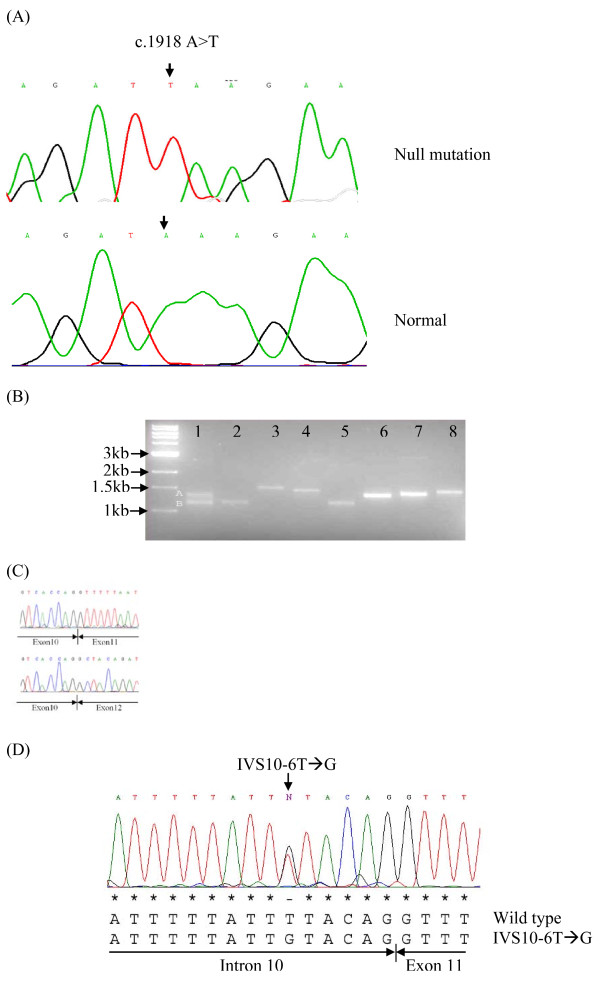
**The 2 ATM truncation mutations identified for low expresser patient 13 are non-allelic**. (**A) **RT-PCR nucleotide sequence chromatogram showing a novel premature termination mutation c.1918A>T: Lys640Stop (arrowed top panel) compared to the normal sequence (lower panel). Note: the upper chromatogram shows no evidence of expression of the normal 'A' nucleotide from the alternate allele. (**B) **RT-PCR amplification of the ATM cDNA for patient 13 using 8 sets of overlapping primers (supplementary table 1) returned single amplicons (lanes 2-8) with the exception of Fragment 1 (lane 1) which returned a dual amplicon suggestive of a deletion in one allele. (**C**) RT-PCR nucleotide sequence chromatograms for these two amplicons; sequence from the normal allele (upper panel) and the shorter allele (lower panel). Note: the shorter allele is missing sequence from exon 11. (**D**) Genomic sequencing revealed a heterozygous variation IVS10-6T-->G (1065del164) within the extended 5' splice site of intron 10 (ATM Ref Seq. NM_000051). Extended RT-PCR sequencing across both mutations confirmed these two mutations were non-allelic (result not shown).

Patient 13 had the lowest ATM level of ~8% compared to the highest expresser (Fig. 2 and see also Fig. 1C lanes 5 & 6). This very low basal level of the protein increased marginally following exposure to IR (Fig. 1C and Fig. 3A &3B) comparable with the degree of IR-induction of ATM protein level observed for the highest expresser (Fig. 1C lanes 7 & 8). Note: the role of this apparent stabilisation of the ATM protein following exposure to IR is uncertain [15], whereas, the IR-induction of ATM kinase activity is a direct, but not quantitative, measure of ATM function (see section 3.4).

### 3.3 Nucleotide Sequence Analysis

In the first instance we used RT-PCR to determine the nucleotide sequence of the protein coding region of the ATM transcript for the 4 low expresser patients. We identified ATM point mutations for two of the low expressers, patients 10 and 13. Patient 10 had a novel missense mutation c.5557G>A:Asp1853Asn which was confirmed by genomic sequencing of exon 39 (result not shown). For patient 13 we identified a novel premature termination mutation c.1918A>T: Lys640Stop (Fig. 3A) which was confirmed by genomic sequencing of exon 15. In addition, the RT-PCR amplification of fragment 1 for patient 13 was abnormal in that it generated a dual amplicon (lane 1 Fig. 3B). To investigate this further, we compared the nucleotide sequence for both the small and large amplicons and found that the smaller amplicon was missing the nucleotide sequence for exon 11 (Fig. 3C). Further genomic DNA sequencing in that region spanning the intron-exon boundaries adjoining exon 11 identified a heterozygous mutation c.1066-6T>G (IVS10-6T>G) within the extended 5' splice consensus sequence of intron 10 (Fig. 3D). This mutation generated an aberrant splice site recognition sequence r.ex11del (1065del164) with potential to effect skipping of exon 11. This mutation has been reported previously in women with breast cancer [28]. Splicing mutations outside the canonical AG-splice acceptor site and GT splice donor sites are relatively common in A-T patients [16] and ATM exon skipping has been reported previously for A-T families including skipping of exons 4, 6, 7, 8, 9, 10, 11, 12, 14 or 16 http://chromium.liacs.nl/lovd/refseq/ATM_codingDNA.html[29]. To determine the allelic status of these two mutations in patient 13 we employed a RT-PCR strategy that spanned both mutation sites within a single amplicon ie. by using primers complimentary to sequence from exon 10 and exon 15, respectively. Nucleotide sequence of the different sized amplicons indicated that the two ATM mutations for patient 13 were not allelic but located on different chromosomes. The RT-PCR sequence chromatogram for the c.1918A>T mutation (Fig. 3A) further indicated a reduced level of that allele that skips exon 11, presumably the result of nonsense mediated decay.

For low expressers 11 and 16 there was no evidence for a mutation in the ATM coding sequence. Patient 11 had a higher than normal ICA count (see below) and an unusual ATM kinase activity profile, therefore, we performed a full genomic sequence analysis of the ATM gene for this patient spanning the intron-exon boundaries for all 66 exons. We found no evidence for a deleterious mutation in the ATM gene for patient 11.

### 3.4 ATM Kinase Activity

**Figure 4:**
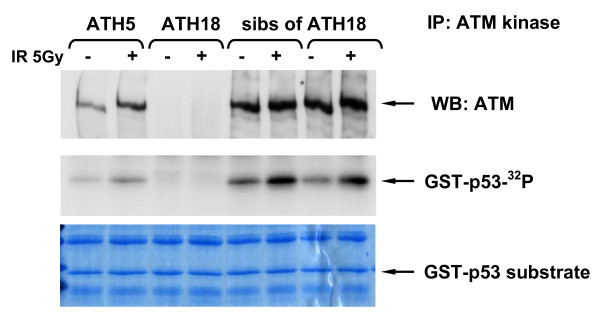
**ATM kinase activity for LCLs 1 hour after an *in vitro *IR exposure of 5 Gy for breast cancer patients and controls**.

**Figure 5:**
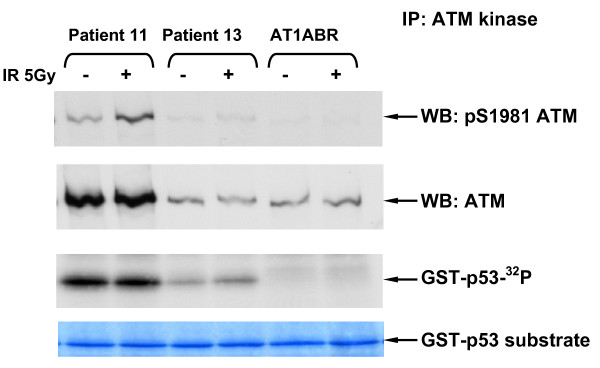
**ATM kinase activity for LCLs 1 hour after an *in vitro *IR exposure of 5 Gy for breast cancer patients and controls**.

**Figure 6:**
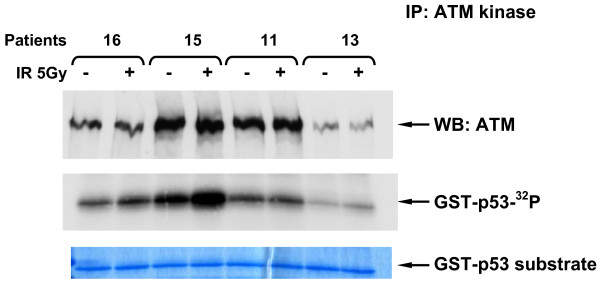
**ATM kinase activity for LCLs 1 hour after an *in vitro *IR exposure of 5 Gy for breast cancer patients and controls**.

**Figure 7:**
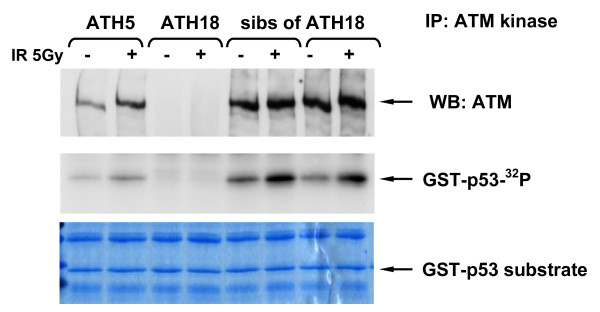
**ATM kinase activity for LCLs 1 hour after an *in vitro *IR exposure of 5 Gy for breast cancer patients and controls**. Obligate heterozygote ATH5ABR ~ the father of an A-T patient and his two siblings had high ICA counts of 2.18, 3.24, 2.50 & 2.48, respectively. The AT1ABR cell line, used here as a positive control, was originally derived from an A-T patient homozygous for a small (3 amino acid) in-frame deletion that destabilises the protein and which was reported previously with very low levels of mutant ATM protein [18].

To determine the functional significance of low levels of the ATM protein we tested all low expressing patient cell lines for ATM kinase activity [20] and IR-induced chromosomal aberrations (ICA). *In vitro *ATM kinase activity was measured using ATM immunoprecipitation as described previously [18] followed by immunoblotting with the anti-pS1981 antibody to determine autophosphorylation, and also by using p53 as a substrate for ATM (Figs. 4, 5 &6). We found that 3/4 of the low expressers (patients 11, 13 & 16) had reduced ATM kinase activity (Figs. 4, 5, 6 &7 and reviewed in Table 1). Patient 16 showed no detectable level of ATM kinase activity (Fig. 6). The very low level of ATM protein for patient 13 (Fig. 4, 5, 6) was associated with an equally low level of ATM autophosphorylation. This was confirmed using the GST-p53 substrate which showed a small level of radiation-induced phosphorylation that was only evident at the higher dose of 10 Gy (Fig. 4, lower panels), see longer exposure. After immunoprecipitation, low expresser patient 11 also showed a small degree of ATM autophosphorylation, however, this induction of ATM kinase activity was not evident when p53 was used as the substrate (Fig. 5). Low expresser patient 10 (35% of the ATM protein) showed no reduction in kinase activity which suggested a possible false positive status, however, ATM kinase assays are not strictly quantitative. The only A-T obligate heterozygote in this study to carry a missense mutation, ATH5ABR, had the lowest level of the ATM protein (17%) when compared to the other ATH (Fig. 2 and see also Fig. 1A lane 10 and Fig. 7) and low ATM kinase activity (Fig. 7). A larger study could help here to determine the stoichiometric relationship between ATM protein level and detectable reductions in kinase activity.  The A-T affected child of ATH5ABR (ATH18ABR) had no ATM protein and no ATM kinase activity (Fig. 7).

### 3.5 IR-induced chromosomal aberrations (ICA)

The number of DNA double strand breaks that persist following exposure to IR *in vitro *(chromosomal aberrations) is a relative measure of any inefficiency in dsDNA damage repair. ICA counts > 1.25 per metaphase are indicative of a deficit in DNA repair [21]. In this study the mean for normal controls was < 1. The two A-T cell lines used here as ATM null controls had mean levels of ICA > 3. The 4 low expresser patients (patients 10, 11, 13 and 16) had ICA counts of 1.44, 1.34, 1.52, and 0.94, respectively (Table 1). The ICA count for ATH5ABR was 2.3. ICA counts in this study correlated more consistently with clinical radiosensitivity than with *in vitro *cellular proliferation assays following IR (Table 2) or clonogenic assays (results not shown) [25]. For example, low expresser patient 13, the patient with the compound pathogenic ATM genotype and the lowest level of ATM protein (~10%) and very low ATM kinase activity and a high ICA count had an average *SF_2 _*value of 41.6 which was higher than the other 3 low ATM expresser patients 10, 11 and 16 (22.4, 25.7 and 33.8, respectively) and comparable to that for non-reactor controls (39.5 ± 5.98) and higher compared with the *SF_2 _*value for women with SLE (33.0 ± 3.19) (Table 2).

**Table 2:** LCL survival fractions following exposure to 2 Gy IR

Patients	SLE	Non-Reactor Controls	Low ATM Expressers	A-T
**Average SF_2 _(%)**	30.40	37.50	23.1 (10)	17.2
	36.55	38.62	25.7 (11)	9.3
	32.08	48.83	41.6 (13	
	30.88	36.65	33.8 (16	
	32.98	45.51		
	36.80	31.21		
	33.42	39.11		
	29.46	35.45		
	34.45	33.96		
	34.83			
	35.18			
	30.25			
	27.18			
	38.35			
				
**Mean SF_2_**	**33.0 ± 3.19**	**39.5 ± 5.98**		

## Discussion

Informative radiotherapy biomarkers have great potential to improve risk assessments and radiotherapy outcomes. The pathological risk for clinical radiosensitivity and/or breast cancer associated with ATM mutations is considered to be low but in most risk assessments this risk could better be described as uncertain due to the omission of functional data [9][11][12]. Few of the ATM variants/mutations reported to date have been functionally characterised and the common omission of functional data from ATM related risk assessments [9,11,12] is disconcerting especially if the data is not supportive or conclusive. Albeit, there is uncertainty regarding the pathological threshold level of the ATM protein and the extent to which ATM protein levels vary independent of ATM mutation status.

Severe reactions to radiotherapy are very rare entities and our cohort of 20 radio-hypersensitive individuals represented patients recruited from 3 of Australia's larger cancer centres. A total of 12 members of this cohort had been treated for breast cancer and 4 of this 12 had levels of the ATM protein < 55% compared with the mean for non-reactor controls. Subsequently all 4 of these 'low ATM expressers' were found to have either reduced ATM kinase activity (3/4) and/or increased ICA counts (3/4) and/or ATM mutations (2/4) (summarised in Table 1). Note: The study of the cellular stress response to IR reported here may bring forward a cellular phenotype for some individuals including some A-T heterozygotes where an early age at presentation would otherwise be unexpected. A-T patients (ie, homozygotes with the clinical syndrome) present at a young age, whereas obligate A-T heterozygotes usually remain undetected and are generally asymptomatic except for risk of breast cancer and radiosensitivity. A larger study including A-T families could help refine ATM protein thresholds for risk of both clinical radiosensitivity and breast cancer.

The persistence of ICA in 3/4 of the low expressers provided a particularly clear link with the clinical radiosensitivity for these patients, two of whom manifest both severe acute and severe late reactions. Of these 2 patients, patient 11, had a high ICA count and low ATM kinase activity but no ATM mutation at either the cDNA or genomic level suggesting that ATM protein levels may represent an independent risk factor for clinical radiosensitivity [30-33]. Compared to ICA, cellular proliferation assays in this study were not as reliable an indicator of clinical radiosensitivity due to variation in cell cycling parameters and where some LCLs demonstrate markedly reduced and often variable growth rates after replating and/or variable growth rates at different cell densities. The increased complexity and extended duration of proliferation assays can further compound these variations.

Greater knowledge regarding the functional deficiency associated with any ATM mutation will help clarify the associated pathological risk. The present study demonstrates that levels of haploinsufficiency associated with a range of ATM null mutations do vary between individuals (Fig. 2). Results further suggest that haploinsufficiency is a risk factor only when the associated level of protein falls below a threshold for breast cancer or clinical radiosensitivity. From the results of this small pilot study we propose a tentative ATM threshold of ~55% for risk of clinical radiosensitivity for breast cancer patients compared to the mean for non-reactor controls. Haploinsufficiency can also result from missense mutations. The obligate A-T heterozygote ATH5ABR who carried what could have been regarded as a 'low-risk' missense variant (Leu950Arg) [12] due to its location outside the evolutionary conserved domains of ATM to which missense variants have been most closely tied to A-T (namely the FAT, kinase and FATC domains located at amino acid positions 2096-2489, 2711-2962 and 3024-3056, respectively within ATM [12,27,34]) was associated with a very low level of the ATM protein (~17%), reduced ATM kinase activity and a high ICA count [13] to provide the strongest indication of pathogenic potential. Results also suggest that individuals carrying the same mutation may express different levels of the ATM protein. For example, radiosensitive breast cancer patient 13 had a non-allelic compound pathogenic ATM genotype comprised of a truncating Lys640Stop mutation on one allele and on the other allele a splice site mutation c.1066-6T > G (that caused skipping of exon 11 and premature termination in exon 12 sequence) that reduced transcript stability and the protein level (~10%) but did not result in any of the more severe early-onset A-T pathologies. In contrast, homozygosity for this same splice site mutation was reported previously in an A-T patient with < 10% of the full length ATM protein [29] indicating the 'leaky' nature of this mutation and suggesting variable expressivity between individuals. The coexistence of pathological thresholds and variable expressivity of ATM protein levels presents opportunities and pitfalls for future risk assessments that could best be managed with the determination of ATM protein levels for all subjects regardless of mutation status.

Do ATM protein levels represent a practical biomarker for assessing risk of radiotherapy toxicity or breast cancer? Unlike the ATM kinase assay, ATM protein quantitation is quantitative yet at this point in time both assays require large cell numbers. With modern advances in mass spectrometry [22,24] it may soon be possible to evaluate ATM levels in single cell analyses - a development that would greatly expedite testing in the clinic. There may also be future scope to determine tumour thresholds for ATM for optimum IR control of cancer. The 4 'low ATM expressers' described here with breast cancer did not recur within the 2-5 year period following radiotherapy. In addition, low ATM protein levels are usually associated with more advanced stages of breast cancer [15,35] with a notable exception being the high level expression of ATM in a radioresistant malignant myoepithelioma of the breast [36] which together suggest that there may exist an ATM threshold for optimum radiotherapy control.

## Conclusion

ATM mutations are generally considered low risk alleles for breast cancer and clinical radiosensitivity. Results described here suggest that ATM protein levels may represent an independent biomarker for individuals at high-risk of clinical radiosensitivity. We propose a tentative ATM threshold of ~55% for risk of adverse normal tissue reactions to radiotherapy and ~10% for the more severe A-T related pathologies.

## Competing interests

The authors declare that they have no competing interests.

## Authors' contributions

ZF - carried out radiosensitivity studies and molecular genetic studies

SK - carried out molecular genetic studies, ATM kinase assays and helped draft manuscript

MM - performed clinical analysis and helped draft manuscript

RW - carried out molecular genetic studies

GB - carried out molecular genetic studies

CS - carried out molecular genetic studies

DM - performed clinical analysis

KW - carried out protein analysis

LT - performed sequence analysis

JK - Head Clinician and conceived study

ML - helped with study design and draft manuscript

PG - performed clinical analysis and helped draft manuscript

RC - Designed the study and participated in its coordination, carried out molecular genetic studies and drafted the manuscript

All authors read and approved the final manuscript.
